# Supplementary figures and images for: Thrombotic microangiopathy after kidney transplantation: Analysis of the Brazilian Atypical Hemolytic Uremic Syndrome cohort
Source: PLoS One. 2021 Nov 8;16(11):e0258319. doi: 10.1371/journal.pone.0258319 (PMC8575299; doi:10.1371/journal.pone.0258319)

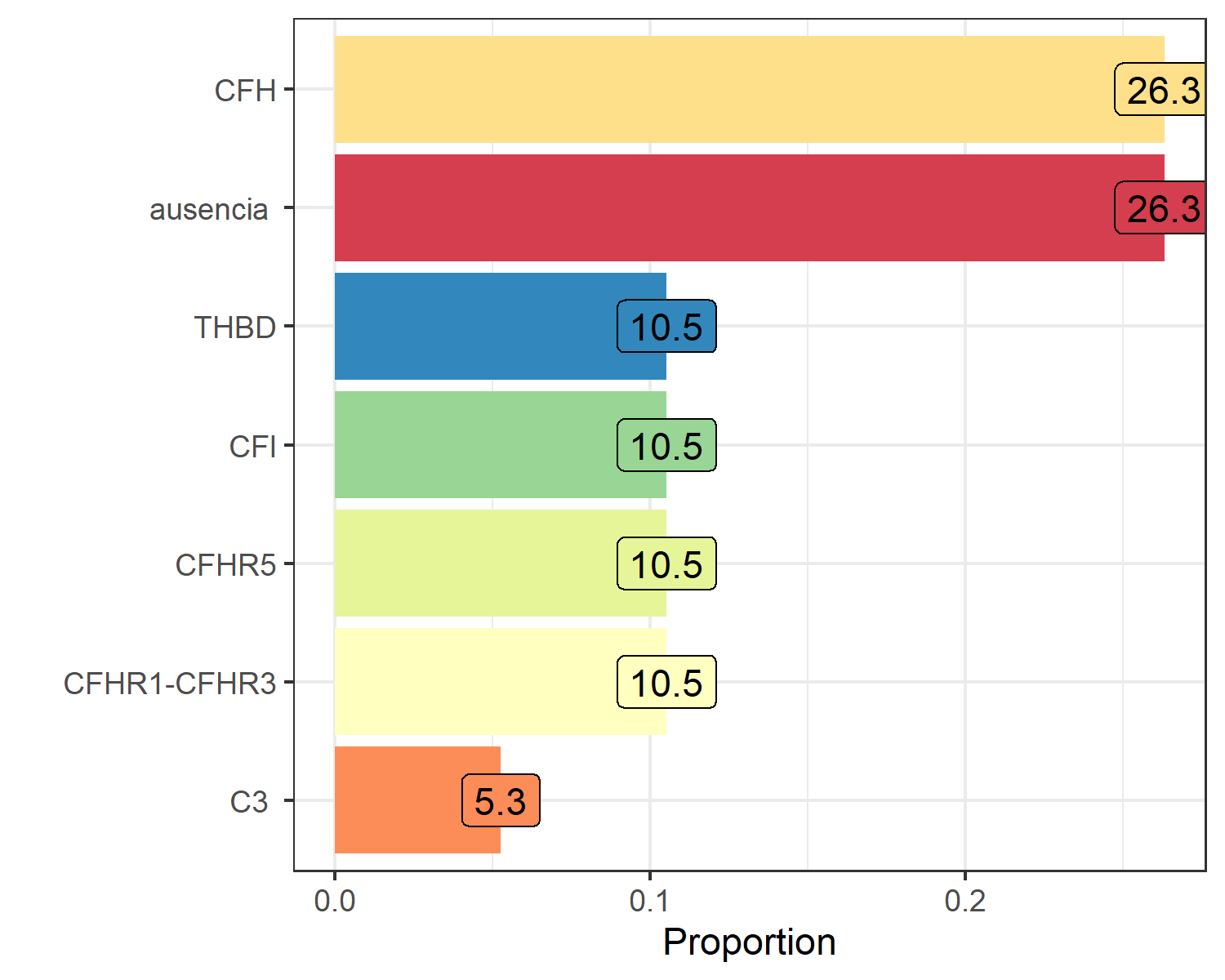

Supplement: S1 Fig — (TIF) [file pone.0258319.s001.tif]
