# Supplementary material for: Thrombotic microangiopathy after kidney transplantation: Analysis of the Brazilian Atypical Hemolytic Uremic Syndrome cohort
Source: PLoS One. 2021 Nov 8;16(11):e0258319. doi: 10.1371/journal.pone.0258319 (PMC8575299; doi:10.1371/journal.pone.0258319)
Supplement: S1 Table — (DOCX) [file pone.0258319.s002.docx]

**S1 Table. The demographic data and complete genetic analysis in the Brazilian aHUS cohort in kidney transplantation.**

| Id | Sex | Age  (Years) | Underlying Kidney Disease | Genetics | Detail Genetic Analysis | Classification |
| --- | --- | --- | --- | --- | --- | --- |
| 1 | Male | 58 | C3 Nephropathy | without variants |  | without variants |
| 2 | Male | 55 | C3 Nephropathy | not performed |  |  |
| 3 | Female | 24 | aHUS | not performed |  |  |
| 4 | Male | 32 | Indeterminate | C3 | Heterozygous variant C3 c.463A>C p.(Lys155Gln) | Class 2- likely pathogenic |
| 5 | Female | 29 | Indeterminate | CFH | heterozygous variant CFH c.2056+ 1G>A | Class 2- likely pathogenic |
| 6 | Female | 29 | aHUS | CFH | heterozygous variant CFH c.2056+ 1G>A | Class 2- likely pathogenic |
| 7 | Male | 31 | Indeterminate | not performed |  |  |
| 8 | Male | 19 | aHUS | CFH | variant CFH c.3148A>T p.(Asn1050tyr) | Class 3-variant of unknown significance |
| 9 | Male | 21 | aHUS | CFH | variant CFH c.3148A>T p.(Asn1050tyr) | Class 3-variant of unknown significance |
| 10 | Female | 38 | Indeterminate | without variants |  | without variants |
| 11 | Female | 31 | Indeterminate | not performed |  |  |
| 12 | Female | 17 | aHUS | CFHR5 | Heterozygous variant CFHR5 c.1067G>A (p.Arg356His) | Class 2- likely pathogenic |
| 13 | Female | 45 | Indeterminate | without variants |  | without variants |
| 14 | Female | 45 | aHUS | not performed |  |  |
| 15 | Male | 35 | Indeterminate | CFI, CFB | Heterozygous variant CFI c.[1246A>C(;)1071T>G]; p.[Ile416Leu) (;) (Ile357Met)], Heterozygous variant CFB c.1505T>C;p.(Ile502Thr | Class 2- likely pathogenic |
| 16 | Male | 33 | Indeterminate | without variants |  | without variants |
| 17 | Female | 48 | Indeterminate | not performed |  |  |
| 18 | Male | 32 | Glomerulonephritis | CFI,CFHR1,CFHR3 | Heterozygous varint CFI c.1246A>C(p.lle416Leu),  Heterozygous deletion encompassing the entire gene CFHR1 e CFHR3 | Class 3-variant of unknown significance |
| 19 | Female | 45 | Glomerulonephritis | not performed |  |  |
| 20 | Female | 16 | aHUS | CFH, CFI | Heterozygous variant CFH c.3628C>T p.(Arg1210Cys),  Heterozygous variant CFI c.502A>G p.(Arg168Gly) | Class 1-pathogenic (CFH ) and variant of unknown significance (CFI) |
| 21 | Female | 19 | aHUS | CFHR5, THBD | Heterozygous variant CFHR5 c.432A>T;p.(Lys144Asn),  Heterozygous variant THBD c.40G>A;p.(Gly14Ser) | Class 3-variant of unknown significance |
| 22 | Female | 26 | Indeterminate | not performed |  |  |
| 23 | Female | 30 | aHUS | not performed |  |  |
| 24 | Female | 36 | aHUS | CFHR5, CFHR1 | Heterozygous variant CFHR5 c.254-2_266dup p.(Ser88_Phe89ins-LeuGlyMetCysSer)/ CFHR1 (MLPA) | Class 2- likely pathogenic |
| 25 | Male | 29 | Indeterminate | not performed |  |  |
| 26 | Female | 26 | Indeterminate | not performed |  |  |
| 27 | Female | 27 | Indeterminate | not performed |  |  |
| 28 | Female | 29 | aHUS | not performed |  |  |
| 29 | Female | 42 | Glomerulonephritis | not performed |  |  |
| 30 | Female | 45 | Indeterminate | not performed |  |  |
| 31 | Female | 29 | Glomerulonephritis | CFHR1 e CFHR3 | Heterozygous deletion encompassing the genes CFHR1 e CFHR3 | Class 3-variant of unknown significance |
| 32 | Male | 29 | Glomerulonephritis | not performed |  |  |
| 33 | Male | 18 | Indeterminate | not performed |  |  |
| 34 | Female | 23 | Indeterminate | not performed |  |  |
| 35 | Female | 45 | Indeterminate | CFHR1 e CFHR3 | Homozygous deletion encompassing the genes CFHR1 e CFHR3 | Class 2- likely pathogenic |
| 36 | Male | 40 | Indeterminate | not performed |  |  |
| 37 | Female | 21 | Indeterminate | not performed |  |  |
| 38 | male | 20 | aHUS | THBD, PLG | Heterozygous variant THBD c.1208G>a p. (Arg403Lys)/ Heterozygous variant PLG c.758g>A p.(arg253His) | Class 3-variant of unknown significance |

Legends: aHUS: Atypical Hemolytic Uremic Syndrome
