# Supplementary material for: Thrombotic microangiopathy after kidney transplantation: Analysis of the Brazilian Atypical Hemolytic Uremic Syndrome cohort
Source: PLoS One. 2021 Nov 8;16(11):e0258319. doi: 10.1371/journal.pone.0258319 (PMC8575299; doi:10.1371/journal.pone.0258319)
Supplement: S2 Table — (DOCX) [file pone.0258319.s003.docx]

**S2 Table. The frequency of variants divided by groups: no eculizumab use, eculizumab treatment, and prophylactic eculizumab in the Brazilian aHUS cohort in kidney transplantation.**

| Groups |  |  |  |  |
| --- | --- | --- | --- | --- |
|  | **Id** | **Classification** | **% total** | **% with genetic analysis** |
| No treatment | 5 | Class 2- likely pathogenic | Class 2: 2/11: 18% | 2/5:40% |
|  | 35 | Class 2- likely pathogenic |  |  |
|  | 8 | Class 3-variant of unknown significance | Class 3: 2/11: 18% | 2/5:40% |
|  | 31 | Class 3-variant of unknown  significance | |  |
|  | 1 | without variants | without variants: 1/11:9% | 1/5: 20% |
|  | 2 | not performed |  |  |
|  | 19 | not performed |  |  |
|  | 22 | not performed |  |  |
|  | 25 | not performed |  |  |
|  | 27 | not performed |  |  |
|  | 36 | not performed |  |  |
|  |  |  |  |  |
| Treatment | 4 | Class 2- likely pathogenic | Class 2: 2/17: 11,7% | 2/6: 33% |
|  | 15 | Class 2- likely pathogenic |  |  |
|  | 9 | Class 3-variant of unknown significance | Class 3: 2/17: 11,7% | 2/6:33% |
|  | 18 | Class 3-variant of unknown  significance | |  |
|  | 10 | without variants | without variants: 2/17: 11,7% | 2/6:33% |
|  | 13 | without variants |  |  |
|  | 7 | not performed |  |  |
|  | 11 | not performed |  |  |
|  | 17 | not performed |  |  |
|  | 26 | not performed |  |  |
|  | 28 | not performed |  |  |
|  | 29 | not performed |  |  |
|  | 30 | not performed |  |  |
|  | 32 | not performed |  |  |
|  | 33 | not performed |  |  |
|  | 34 | not performed |  |  |
|  | 37 | not performed |  |  |
|  |  |  |  |  |
| Prophylactic | 20 | Class 1-pathogenic | Class 1: /10:10% | 1/7:14% |
|  | 6 | Class 2- likely pathogenic | Class 2: 3/10:30% | 3/7: 42% |
|  | 12 | Class 2- likely pathogenic |  |  |
|  | 24 | Class 2- likely pathogenic |  |  |
|  | 21 | Class 3-variant of unknown significance | Class 3: 2/10:20% | 2/7: 28% |
|  | 38 | Class 3-variant of unknown s  ignificance | |  |
|  | 16 | without variants | without variants: 1/10:10% | 1/7:14% |
|  | 3 | not performed |  |  |
|  | 14 | not performed |  |  |
|  | 23 | not performed |  |  |

Legends: aHUS: Atypical Hemolytic Uremic Syndrome
