# Supplementary material for: Thrombotic microangiopathy after kidney transplantation: Analysis of the Brazilian Atypical Hemolytic Uremic Syndrome cohort
Source: PLoS One. 2021 Nov 8;16(11):e0258319. doi: 10.1371/journal.pone.0258319 (PMC8575299; doi:10.1371/journal.pone.0258319)
Supplement: S3 Table — (DOCX) [file pone.0258319.s004.docx]

**S3 Table. Cause of mortality in the Brazilian aHUS cohort in kidney transplantation.**

| Group | Mortality Cause | Time Post-transplant |
| --- | --- | --- |
| Eculizumab treatment | Hemorrhagic shock in the postoperative period | 9 months |
| Eculizumab treatment | Septic shock after hysterectomy and enterectomy | 32 months |
| Eculizumab treatment | Septic shock secondary to an infected foot ulcer | 10 months |
| Eculizumab treatment | Septic shock to urinary infection. | 21 months |
| No Eculizumab use | TMA recurrence | 21 months |
| No Eculizumab use | TMA recurrence | 6 months |

Legends: aHUS: Atypical Hemolytic Uremic Syndrome; TMA: Thrombotic Microangiopathy
