# Supplementary material for: Thrombotic microangiopathy after kidney transplantation: Analysis of the Brazilian Atypical Hemolytic Uremic Syndrome cohort
Source: PLoS One. 2021 Nov 8;16(11):e0258319. doi: 10.1371/journal.pone.0258319 (PMC8575299; doi:10.1371/journal.pone.0258319)
Supplement: Attachment — Submitted filename: reviewrs_response.docx [file pone.0258319.s005.docx]

**Response to Reviewers**

**Reviewer #1:** The authors report on their experience with renal transplantation in patients with a presumed or confirmed diagnosis of aHUS in 6 Brazilian transplant centres. They included 38 patients, a significant number of patients in regard of the rarity of the disease. For the purpose of the analysis, the patients were divided into three groups: no treatment with eculizumab, curative or prophylactic treatment with eculizumab.
The authors conclude that eculizumab use (particularly as prophylaxis) significantly improved graft but also patients’ survival. The novelty of the data is rather limited, and previous larger studies have shown similar trends for graft loss due to aHUS recurrence.
I have the following major comments:

a) The cause of end-stage renal disease is undetermined in a large proportion (almost half) of patients.

**In the initial pre-transplant evaluation, the diagnosis of kidney disease was undetermined. However, at this time, the aHUS evaluation was not carried out because they had no diagnostic suspicion. These patients evolved with aHUS after transplantation and after that were treated with eculizumab (eculizumab treatment group). In fact, these groups of patients were probably aHUS undiagnosed prior kidney transplant.**

b) The complement genetic data are available in only 19/38 included patients and the distribution of complement gene variants within the three groups of patients is unknown. This is a crucial point as complement variants are a major predictor for aHUS relapse after renal transplantation. The detected variants should also be classified as pathogenic, likely pathogenic or variants of unknown significance.

**Thank you for the very important comment. We adjusted the table with this requested information – (supplementary S2 Table and S3 Table).**

c) The high incidence of acute rejection (67%) in the group of patients who did not receive eculizumab is clearly a confounding factor for the analysis of graft loss. Besides, this group included the highest rate of living donors (55%) and no induction therapy in 44% of cases. It is in sharp contrast with the Dutch experience with living donation without prophylactic eculizumab in aHUS patients.

**We agree with the reviewer. The group that did not receive the eculizumab was different from the other two groups. These patients received a kidney transplant in older time and had higher rates of acute rejection and lower use of induction therapy. These may compromise the results of the graft survival analysis. We included these in the limitations. However, we believe that this limitation did not compromise the primary study aim that was the TMA recurrence.**

d) What were the causes of death in patients who did not receive prophylactic eculizumab? A rate of 20-27% of death in two groups of patients is unusually high and hardly explained by a potential aHUS recurrence.

**We considered a broad range of follow-up, and it is possible that the mortality was not exclusively related to aHUS recurrence. We included a one-year death in addition to the all-time follow-up death.**

**One-year death rate was respectively in no eculizumab treatment, prophylactic and eculizumab treatment: 9%, 0% and 12%. We also included a supplementary Table with all causes and time of death (S4 Table)**

| **Group** | **Mortality Cause** | **Time Post-transplant** |
| --- | --- | --- |
| **Eculizumab treatment** | Hemorrhagic shock in the postoperative period | 9 months |
| **Eculizumab treatment** | Septic shock after hysterectomy and enterectomy | 32 months |
| **Eculizumab treatment** | Septic shock secondary to an infected foot ulcer | 10 months |
| **Eculizumab treatment** | Septic shock to urinary infection. | 21 months |
| **No Eculizumab use** | TMA recurrence | 21 months |
| **No Eculizumab use** | TMA recurrence | 6 months |

d) The discussion is more focused on the accessibility to eculizumab in Brazil rather than on the results of the study. A cost-effective use of eculizumab in renal transplantation is based in good part on genetic analysis, and this point needs to be more developed (see reference Zuber et al. JASN).

**We agree and discuss the cost-effective use of eculizumab in renal transplantation based on genetic analysis:**

**The cost-effectiveness of accessing eculizumab for kidney transplant patients should be based on genetic analysis, thus, it is necessary to include the genetic profile for patients with suspicion for aHUS. In those classified at high to moderate risk according to KDIGO 2017 we strongly consider prophylaxis. As the opposite, we consider discontinuing the use in those without identified genetic variants, as well as the saving of 32 million euros realized by the STOPECU study (Fakhouri et al, 2021) after the discontinuation of 55 patients with an average of 24 months of follow-up.**

**Reviewer #2:** This was a multicenter retrospective cohort study on 38 patients with ESKD due to aHUS who underwent kidney transplant. Patients were stratified into three groups: no use of eculizumab (No Eculizumab Group), use of eculizumab for treatment of after transplantation TMA (Therapeutic Group), and use of eculizumab for prophylaxis of aHUS recurrence (Prophylatic Group). In the No Eculizumab Group (n=11), there was a 91% graft loss due to the TMA. The hazard ratio of TMA graft loss was 0.07 [0.01-0.55], p = 0.012 in the eculizumab Prophylatic Group and 0.04 [0.00 - 0.28], p =0.002 in the eculizumab Therapeutic Group. The authors conclude that TMA graft loss in the absence of a specific complement-inhibitor was higher among the Brazilian cohort of kidney transplant patients. This finding reinforces the need of eculizumab use for treatment of aHUS kidney transplant patients.

This is a nice retrospective study in a group of patients with an ultra-rare disease.

Comments:
1. How many patients lost their graph in the Prophylactic and Treatment group? The manuscript just gives %

**We provided the number in the text and Table 03.**

| **Group** | **Lost-Graft** |
| --- | --- |
| **Eculizumab treatment (n=17)** | TMA recurrence (n=1) |
| **Prophylactic eculizumab (n=10)** | TMA recurrence (n=1) |
| **No Eculizumab use (n=11)** | TMA recurrence (n=10) |

2. Please clarify: “42% underwent plasmapheresis and 79% received eculizumab”. This is not clear for total number of patients that received eculizumab was 27/38 = 71%

**That's correct, 71% received eculizumab. We will adjust it.**

3. “TMA graft loss occurred in 32% of cases and 17% died” please give actual numbers.

**We provided the numbers through the text**

4. Please provide details of the genetic analysis for the group treated with Eculizumab versus the group that was untreated.

**We will send it in supplementary (S3 Table)**

5. Table 2. 18 + 19 = 37. There is 1 patient missing.

**Sorry, it was a mistake. There are 19 without testing and 19 with genetic variant analysis**

6. Please provide details of the Eculizumab regime used. Was Eculizumab ever discontinued post-transplant? What was the criteria used? What was the outcome in patients that discontinued Eculizumab?

**No patient was discontinued eculizumab after starting treatment. In the prophylactic group one patient evolved to graft loss due to TMA after hospitalization due to arteriovenous fistula thrombosis and had a delay of eculizumab infusion at that time. In the treatment group one patient lost the graft, probably due to a delay in starting eculizumab treatment, which was performed more than 120 days after diagnosis.**

**We provided these informations in the text**

7. There are a few typos/grammar mistakes that need to be fixed.

**We are sorry, we revised the manuscript for grammar mistakes**

**Reviewer #3:** This interesting article highlights the efficacy of complement blockage in preventing and treating the aHUS recurrence after kidney transplantation. The results are consistent with those previously published by Zuber and Siedlecky, as you mentioned in the paper. It would had been very interesting if the genetic study of all patients was available. In spite of that, is important to remark that the patients with CFI mutations had TMA recurrence only if they have combined mutations

- P26 line 8:
"However, the inclusion of the eculizumab as a therapeutic option after transplantation has been significantly improving graft survival by preventing the recurrence of TMA"
In this line, only prophylactic eculizumab prevents recurrence. Please, clarify.

**We modified to:**

**"However, the inclusion of the eculizumab as a therapeutic or prophylactic option after transplantation has been significantly improving graft survival by preventing the recurrence of TMA"**

- In the discussion section, you have mentioned the probable causes associated to the high rate of acute rejection. However, you haven't showed data related to this assumption in the results section. Could you give more information?

**Unfortunately, we could not retrieve more information about the episodes of acute rejection especially in the cases related to the untreated group (the majority were not biopsy proven). We add this information in the limitations section.**

- The differences in the survival probabilities between groups are not statistically significant, probably due to the small sample size. Please, remark this finding

**We highlight these finding**

**Reviewer #4:** I reviewed the manuscript PONE-D-21-24463 titled "Thrombotic microangiopathy after kidney transplantation: analysis of the Brazilian Atypical Hemolytic Uremic Syndrome cohort". This is a retrospective multicentris small cohort study that included only 38 aHUS KTx.
Because the results are confirmatory in nature the paper deserves not more than a Letter to the Editor.

There are 118 kidney transplant centers across Brazil but only ..... 6 accepted to include their patients in this retrospective study that took place between January 2007 and December 2019. during that period of 12 years how many patients were kidney transplanted across Brazil and how many in these 6 centers?

**According to data from the Brazilian Transplant Association (ABTO) we had about 118 kidney transplant centers in Brazil. However, the majority of these transplant centers did not perform transplants in patients with aHUS or did not use eculizumab treatment. The access to eculizumab in Brazil was through compassionate use or judicialization that may be restricted to a small number of transplant centers. These reduced number of centers (n=6) received aHUS patients referred to other transplant programs to perform these special cases of transplants.**

Were donor-specific alloantibodies (DSAs) looked for at the time of TMA recurrence, i.e., in many patients the cause of ESRD was not aHUS?

**All patients had negative donor-specific alloantibodies (DSAs) at the time of TMA diagnosis as part of the aHUS work-up. The undetermined end-stage renal disease is likely to be attributed to patients who probably already have aHUS, but no initial diagnosis was made.**

Across the 3 groups what were the causes of allograft loss?

**The patients lost the grafts by TMA recurrence.**

| **Group** | **Lost-Graft** |
| --- | --- |
| **Eculizumab treatment (n=17)** | TMA recurrence (n=1) |
| **Prophylactic eculizumab (n=10)** | TMA recurrence (n=1) |
| **No Eculizumab use (n=11)** | TMA recurrence (n=10) |
